# The Interactive Effects of Fruit Intake Frequency and Serum miR-484 Levels as Biomarkers for Incident Type 2 Diabetes in a Prospective Cohort of the Spanish Adult Population: The Di@bet.es Study

**DOI:** 10.3390/biomedicines13010160

**Published:** 2025-01-10

**Authors:** Ana Lago-Sampedro, Wasima Oualla-Bachiri, Cristina Maldonado-Araque, Sergio Valdés, Inmaculada González-Molero, Viyey Doulatram-Gamgaram, Elias Delgado, Felipe J. Chaves, Luis Castaño, Alfonso Calle-Pascual, Josep Franch-Nadal, Gemma Rojo-Martínez, Sara García-Serrano, Eva García-Escobar

**Affiliations:** 1 Centro de Investigación Biomédica en Red de Diabetes y Enfermedades Metabolicas Asociadas (CIBERDEM), Instituto de Salud Carlos III, 28029 Madrid, Spain; 2 UGC Endocrinología y Nutrición, Hospital Regional Universitario de Málaga, IBIMA Plataforma BIONAND, 29590 Malaga, Spain; 3 Facultad de Medicina, Universidad de Malaga, Campus de Teatinos s/n, 29071 Malaga, Spain; 4 Centro de Investigación Biomédica en Red de Enfermedades Raras (CIBERER), Instituto de Salud Carlos III, 28029 Madrid, Spain; 5 Department of Endocrinology and Nutrition, Central University Hospital of Asturias, Health Research Institute of the Principality of Asturias (ISPA), University of Oviedo, 33011 Oviedo, Spain; 6 Genomic and Genetic Diagnosis Unit, INCLIVA Biomedical Research Institute, 46010 Valencia, Spain; 7 Cruces University Hospital, Bio-Bizkaia, Department of Pediatrics, University of the Basque Country (UPV/EHU), European Reference Network on Rare Endocrine Conditions (Endo-ERN), 48903 Barakaldo, Spain; 8 Department of Endocrinology and Nutrition, San Carlos University Hospital of Madrid, 28040 Madrid, Spain; 9 EAP Raval Sud, Catalan Institute of Health, GEDAPS Network, Primary Care, Research Support Unit (IDIAP—Jordi Gol Foundation), 08007 Barcelona, Spain

**Keywords:** type 2 diabetes mellitus, incidence, biomarkers, miR-484, fruit intake, interaction

## Abstract

**Background/Objectives**: Although evidence suggests that miR-484 and several fruit components are involved in glucose metabolism and insulin resistance metabolic pathways, the relationship between serum miR-484 levels and fruit consumption in relation to the risk of Type 2 diabetes (T2DM) remains elusive. The aim of this study was to evaluate the possible association between serum miR-484 levels and fruit intake frequency with the risk of T2DM in the Spanish adult population. **Methods**: 2234 subjects from the Di@bet.es cohort study without T2DM at baseline were studied. Socio-demographic, anthropometric and clinical data were recorded, as well as responses to a questionnaire on habits, including frequency of fruit consumption (daily vs. occasional). T2DM was diagnosed at baseline and after 7.5 years of follow-up. Baseline serum miR-484 levels were measured using real-time qPCR and categorized based on the 25th percentile. Association analyses were performed using logistic regression models adjusted for potential confounders. Interaction effects were evaluated on the multiplicative and additive scales. **Results**: There was no association between miR-484 levels and fruit intake frequency. Categorized miR-484 levels and fruit consumption were inversely and independently associated with the likelihood of incident T2DM. Analysis of the interaction effect suggests the presence of both positive multiplicative and additive interactions between miR-484 categories and fruit consumption frequency. **Conclusions**: Our study demonstrates a protective effect of daily fruit intake and high miR-484 levels regarding the risk of T2DM and supports the nutritional recommendations advocating daily fruit consumption. This study also suggests that the combined effect of low miR-484 levels and occasional fruit intake may increase the risk of T2DM beyond their independent effects.

## 1. Introduction

Type 2 Diabetes Mellitus (T2DM), the most common form of diabetes, is a metabolic chronic and ever-growing disease worldwide even reaching epidemic values in developed countries [1]. Environmental risk factors such as physical inactivity and unhealthy dietary habits, in addition to genetic susceptibility and metabolic factors contribute to the development and progression of the disease [2,3,4,5]. Inflammation and oxidative stress also play a key role in the pathophysiology of T2DM, as well as in other related metabolic disturbances [6]; therefore, strategies to reduce inflammation and oxidative stress may be beneficial in the prevention and treatment of these chronic disorders.

Dietary and lifestyle modification are recommended approaches for the prevention of T2DM and its co-morbidities [7]. In addition to regular physical activity, nutrition is a key element in the management and prevention of T2DM, with researchers focused on determine which dietary components have the most beneficial effects [8,9]. Fruit is an important element of various healthy diet patterns, such as the Mediterranean Diet [10]. Fruit is rich in fiber, antioxidants, vitamins, minerals and phytochemical components, all of which have demonstrated beneficial metabolic properties for glycemic control. These include reducing fatty acid deposition, increasing lipid metabolism, modulating insulin signaling pathways, and addressing factors such as gut microbiota and liver inflammation, among others [11,12,13,14,15]. Among the most commonly consumed fruits in Spain are citrus, such as oranges, mandarins and lemons, which are important sources of flavanones like hesperetin and naringenin. These compounds have been shown to have anti-inflammatory and antioxidant effects that may contribute to the health benefits of the Mediterranean diet [16]. However, the presence of sugar in fruit puts a question mark on the ameliorative properties of fruit in subjects with T2DM. In this regard, although most current preclinical and clinical evidence supports the therapeutic role of several natural fruit components in preventing the onset of T2DM and the progression of diabetic complications [14,15,17,18,19], the relationship between fruit intake and T2DM risk remains unclear. Results from a Japanese Public Health Center-based Prospective Study [20] and the CHANCES study [21], which included cohorts from Europe and the United States, found that although a small beneficial effect of fruit cannot be excluded, fruit intake may not be appreciably associated with the risk of T2DM in the Japanese population [20] or in the overall pooled results of a meta-analysis using multiple cohorts older than 50 years from different countries [21]. In contrast, more recent investigations in Chinese adults have shown a positive association between fruit and vegetable consumption and a reduced risk of T2DM [22,23], consistent with a systematic review and meta-analysis of prospective studies in which fruit and vegetable consumption was inversely associated with the risk of T2DM [24].

Despite substantial improvements in the prevention and treatment of T2DM, the associated incidence and mortality rates remain high, highlighting the need for reliable markers for early detection. MicroRNAs (miRNAs) are small, non-coding RNAs involved in the epigenetic regulation of gene expression [25]. MiRNAs have multiple target genes, and alterations in their expression levels have been reported in association with several diseases, including T2DM [26,27,28]. Various miRNAs have been associated with multiple aspects of T2DM and their potential as therapeutic targets and/or biomarkers for the diagnosis and prognosis of T2DM has been extensively discussed in the literature [26,27,28]. Several studies have shown that specific nutritional/dietary factors could regulate the miRNA expression in different cells/tissues related to obesity, T2DM and associated diseases, with consequent effects on the metabolism [29,30,31]. MiR-484 is typically described as an oncosuppressor miRNA; however, its physiological functions in mammals are multifaceted and involve various metabolic processes [32]. MiR-484-targeted genes play an important role in carbohydrate and lipid metabolism, with relevant effects on inflammation, oxidative stress and mitochondrial function [32,33]. MiR-484 is downregulated in pancreatic beta cells in response to high-glucose treatment [34], while in mouse hepatocytes under endoplasmic reticulum stress conditions, miR-484 downregulation is closely associated with in vitro lipoapoptosis [35]. MiR-484 has been strongly associated with early insulin resistance-related metabolites [36], and it has been suggested that miR-484 may also contribute to the pathogenesis of insulin resistance through mitochondrial mechanism by targeting fission protein 1 [37,38]. Altogether, these findings point to the promising role of miR-484 in the control of insulin signalling, glucose transport, insulin resistance and lipid metabolism, all key metabolic processes for the development of T2DM. However, to our knowledge, no previous studies have investigated the potential of miR-484 as a biomarker for the risk of T2DM.

Considering all the above, the aim of this study was to evaluate whether fruit intake frequency and serum miR-484 levels are associated with the risk of incident T2DM over 7.5 years in the Spanish adult population. Additionally, we wondered if a relationship might exist between these two factors in relation to the development of T2DM. Thus, the present study not only provides relevant information to clarify the not-fully established relationship between fruit intake and the development of T2DM in humans, but also evaluates for the first time the role of circulating miR-484 on the risk of incident T2DM, as well as the possible relationship between microRNA levels and fruit intake frequency in explaining increased risk of developing T2DM.

## 2. Materials and Methods

### 2.1. Study Design, Setting and Population

This is a secondary analysis from the population-based cohort study Di@bet.es epidemiological trial based on a subsample of the original cohort (NCT02542735).

The initial cross-sectional study of Di@bet.es was undertaken in 2008–2010 from a random cluster sampling of the Spanish population [39]. Detailed information on the participant flow chart and methodology of the Di@bet.es cohort study, including cross-sectional and follow-up stages, has been previously described [39,40]. Briefly, the Di@bet.es study was a national, cross-sectional, population-based survey conducted in 2008–2010. A cluster sampling design, including 100 clusters (primary care centers), was used to select participants, resulting in a representative random sample of the Spanish population. Of the eligible adults (≥18 years), 55.8% attended an examination, of whom 9.9% were excluded by protocol (institutionalization, severe disease, pregnancy, or recent delivery), giving a final sample of 5072 adult subjects [39]. The sample size was calculated assuming a diabetes prevalence of 15% of the population, with an error margin of less than 1% [40]. The Di@bet.es cohort was re-evaluated in 2016–17 with a follow-up time of 7.5 ± 0.6 years. All subjects who had completed the baseline study were invited (by letter and/or phone) to attend another clinical examination, from which 725 were identified as having T2DM in the cross-sectional study. As with the cross-sectional study, people with serious illness, pregnancy, recent delivery, lactation or surgery within the previous month were excluded. Ultimately, 2408 subjects without T2DM at baseline completed the follow-up. For the present study, only follow-up participants who did not have T2DM at baseline, had nutritional data available, and for whom mir-484 serum levels could be determined were included in the analyses (n = 2234). This sample size was sufficient to detect as significant an OR for developing T2DM of 1.6, with a statistical power greater than 80% at the 95% confidence level, considering a proportion of exposed/unexposed participants of 1/3 and a cumulative incidence of 6.4% in this population [40], as calculated using the OpenEpi website (www.openepi.com).

This research was carried out in accordance with the Declaration of Helsinki (WHO 2011). Written informed consent was obtained from all the participants. The study was approved by the Ethics and Clinical Investigation Committee of the Hospital Regional Universitario de Málaga, Spain (Codes CEI20070612, date 12 June 2007; CEI20110324, date 24 March 2011; CEI20161026 date 26 October 2016; CEI21022019 date 21 February 2019) in addition to other regional ethics and clinical investigation committees all over Spain.

### 2.2. Data Collection and Laboratory Measurements

Participants were invited to attend to a single examination visit at the health center with a nurse specially trained for both phases of this project. A structured questionnaire provided to the interviewer was used to collect information. A physical examination and a blood sample collection were also performed. Nutritional information was determined by means of a 40-item consumption frequency questionnaire [41].

For the present study the anthropometric and socio-demographic variables considered were: age, sex, weight, height, family history of T2DM; educational level (as unlettered, attendance at primary or high school, and university); alcohol consumption (never: no alcohol consumption, low: <1 serving/week, moderate: between 1 serving/week and 2 servings/day for men and 1 serving/day for women, and high: >2 servings/day for men and over 1 serving/day for women); physical activity (classified as low, moderate and high levels according to the Short Form of International Physical Activity Questionnaire Score (SF-IPAQ) [42]); smoking habits (current smokers vs. former/never been smokers). Subjects were asked about their usual frequency of consumption of both whole fruits and homemade 100% fruit juice; data for the previous 6 months were recorded. Frequencies of consumption were reported on an incremental scale with eleven levels (never or almost never, once per month, 2–3 times per month, once per week, 2–3 times per week, 4–6 times per week, once per day, twice per day, 3 times per day, 4 times per day, and 5 or more times per day). Results were finally recoded in two categories: daily (more than or equal to once per day) and occasional (less than once per day).

Blood pressure levels, fasting serum levels of glucose (FGL), insulin and blood lipids were considered, among other clinical variables.

BMI as weight/height^2^ was calculated. Insulin resistance was estimated by the homeostasis model assessment (HOMA) [43], and the insulin resistance risk category (HOMA-IR) was calculated as the HOMA 75th percentile of our population, excluding subjects with T2DM.

### 2.3. Definition of New Cases of T2DM

New cases of T2DM were diagnosed at follow-up according to the presence of at least one of the following:

Fasting serum glucose equal to or higher than 126 mg/dL, 2 h post-oral glucose tolerance test equal to or higher than 200 mg/dL, HbA1c equal to or higher than 6.5%, or use of glucose-lowering medication at the time of examination [44].

### 2.4. miR-484 Level Determination

Total miRNAs from serum samples were isolated using automated methods. MicrocDNA was obtained by reverse transcription using the Universal cDNA Synthesis Kit (Exiqon A/S, Vedbaek, Denmark). Measurements of miR-484 expression levels were performed by real time qPCR using the specific LNA™ PCR primer set (Exiqon A/S, Vedbaek, Denmark) in a Light Cycler 480 (Roche Diagnostics S.L, Barcelona, Spain) at the Genomics platform of the Biomedical Research Institute of Malaga. Quantification of relative miRNA levels was performed using the delta Ct method. MiR-181 was selected as the normalizer miRNA, as it was the most stable in our population, as analysed by the online tool RefFinder (Currently: https://www.ciidirsinaloa.com.mx/RefFinder-master/, accessed on 30 June 2023). Interplate and data normalization were performed by the inclusion of negative controls and a specific calibrator in all plates. Serum levels of miR-484 were analyzed as a quantitative variable to assess differences based on fruit intake frequency and were also categorized according to the 25th percentile (low and high levels) for association analyses related to T2DM development.

### 2.5. Statistical Analysis

Data in tables are presented as mean ± SD, proportions or odd ratios (95% confidence interval). Variables not following a normal distribution were log-transformed to perform the ANOVA analyses. Differences in log-transformed levels of miR-484 according to the fruit intake frequency were measured by ANOVA adjusted by sex, age and BMI. Differences in baseline variables according to miR-484 categories were measured by ANOVA adjusted for sex, age and BMI or Chi-squared test. Association of miR-484 categories and fruit intake frequency with the development of T2DM in the follow-up study was calculated using logistic regression models adjusted for different potential confounders (age, sex, BMI, fasting glucose levels, family history of T2DM, HOMA-IR, physical activity, alcohol consumption and educational level). A *p*-value < 0.05 (two-tailed) was considered statistically significant. All statistical analyses were performed using R software (RStudio 2023.12.1 + 402 “Ocean Storm”).

The InteractionR package for RStudio was used for full reporting of effect modification and interaction analysis. The multiplicative interaction effect was assessed by including the product term of miR-484 categories and frequency of fruit consumption in the adjusted regression models. The relative excess risk due to interaction (RERI) was estimated as a standard measure for the effect measure modification on the additive scale [45]. The attributable proportion (AP) and synergy index (SI) were also estimated to assess the proportion of disease in the highest-risk group attributable to the interaction between the two factors, and to quantify the excess risk from both factors when a biological interaction exists, beyond the risk from both exposures independently [45,46]. RERI or AP scores equal to 0 indicates the absence of additive interaction [47]. Since measures quantifying interaction on an additive scale were developed for use with exposures as risk factors rather than preventive factors, the lowest-risk group was used as the reference in these analyses [47]. Relevant indicators for additive interaction were estimated according to Knol M [47], and their confidence intervals were calculated using the better performing MOVER method introduced by Zou [48]. Finally, an indication of a positive biological interaction was considered when RERI  >  0, AP  >  0, S  >  1, or as a measure of multiplicative interaction for risk ratios  >  1.

## 3. Results

### 3.1. Baseline Characteristics of the Population According to the miR-484 Categories

The study sample included 2234 individuals with a mean age of 48 years (age range 18–89 years), from which 39.2% were men. MiR-484 expression levels ranged between 0.02 and 235.6 (median of 3.92). Percentile 25 of the miR-484 level distribution were calculated and used to define the categories of low (miR-484 levels ≤ 1.65; n = 555) and high (miR-484 levels > 1.65; n = 1679) miR-484.

Differences in baseline characteristics according to the miR-484 categories are presented in Table 1 adjusted by sex, age and BMI, as appropriate. Subjects in the low miR-484 category presented lower BMI than those in the high mir-484 category. The same behavior was found in fasting serum glucose and insulin levels, as well as in HOMA index. Educational levels were also different according to the miR-484 categories.

### 3.2. Relationship Between miR-484 Levels and Fruit Frequency Intake

Frequency of fruit intake did not vary across the miR-484 categories (Table 1). Alternatively, log-transformed miR-484 levels for occasional fruit intake were 0.62 ± 0.54, while for daily fruit intake, log-transformed serum miR-484 levels were 0.57 ± 0.53. Analysis of the differences in these serum levels by general linear models adjusted by sex, age and BMI returned no significant relationship between the variables (*p* = 0.20).

### 3.3. miR-484 as T2DM Development Biomarker

Proportions of subjects who developed T2DM according to the miR-484 categories in the overall population and stratified by frequency of fruit consumption were studied.

In our population, 143 (6.4%) people developed T2DM after 7.5 years of follow-up, among them, 39 (7.1%) reported occasional fruit intake at baseline. In the overall population, no differences were found in the proportions of subjects who developed T2DM according to miR-484 categories (Low miR-484: 7.4%, n = 41 vs. High mir-484: 6.1%, n = 102; *p* = 0.32 measured by Chi-Square test).

When the population was stratified by fruit intake frequency, the percentage of incident T2DM was higher in the group with low miR-484 and occasional fruit consumption (10.4%, n = 13) compared to those low miR-484 but daily fruit consumption (6.5%, n = 28) or those in the high miR-484 category (high miR-484 + occasional fruit intake: 6.1%, n = 26; high miR-484 + daily fruit intake: 6.0%, n = 76). However, these differences in T2DM incidence rates did not reach statistical significance, as measured by Chi-Square test.

The results from the adjusted logistic regression analysis for the development of T2DM at follow-up (after 7.5 years) showed that both high miR-484 and daily fruit intake, were independent protecting factors for the development of T2DM (Table 2). A significant Wald parameter was also found in our models, suggesting that miR-484 and the frequency of fruit consumption had a multiplicative interaction on the risk of T2DM (Table 2). Multivariable analyses for the risk of T2DM considering the frequency of consumption of other foods as covariate were also performed with non-significant results and without altering the relationships presented in Table 2.

The results of the additive interaction analyses are presented in Table 3. As RERI, AP and SI are difficult to interpret when effects are protective [47], the reference category in these analyses was miR-484_High with daily fruit intake as the lowest-risk category. The data reported in Table 3 suggests that the likelihood of developing T2DM was more than four times higher in subjects who consumed fruit occasionally and had low miR-484 levels than in those who consumed fruit daily and were included in the miR-484_High category. The RERI and AP values significantly above 0 (Table 3) showed a positive additive interaction between miR-484 and fruit consumption on the likelihood of T2DM at follow-up; specifically, lower miR-484 category and occasional fruit intake act synergistically to increase the risk of developing T2DM in relation to miR-484_High with daily fruit consumption. According to the interpretation of AP values, 66–67% of the disease in the highest-risk group is due to the interaction between the two factors. However, although SI values above 1 also support a positive interaction, they were not statistically significant.

## 4. Discussion

Different miRNAs have been associated with multiple aspects of T2DM and the potential role of some of them in the development of T2DM has been previously discussed [26,27,28]. To the best of our knowledge, this is the first study investigating the association between serum miR-484 levels and incident T2DM to date. Our results showed that subjects with low miR-484 levels at baseline had a higher probability of developing T2DM compared to those with higher miR-484 levels, independent of traditional T2DM risk factors such as age, sex and baseline fasting serum glucose level or BMI, among others. The possible mechanisms underlying this protective role could be related to previous studies that suggest the promising role of miR-484 in the control of different metabolic processes related to the development of T2DM. It is known that miR-484 targets have relevant effects on inflammation, oxidative stress and mitochondrial function, and GO and Kegg pathway analyses have shown that miR-484 target genes play relevant roles in carbohydrate and lipid metabolism [32,33]. MiR-484 has been reported to be downregulated in pancreatic beta cells in response to high glucose treatment, suggesting that elevated glucose levels associated with insulin resistance may influence circulating miR-484 levels [34]. It has been proposed that down-regulation of miR-484 may also contribute to the pathogenesis of insulin resistance by targeting fission protein 1 [37,38], which is required for mitochondrial fission. As mitochondrial fission is increased in diabetes and contributes to circulating insulin levels [4,5], fission protein 1 expression mitigated by elevated miR-484 levels may lead to reduced glucose metabolism deregulation [32,37]. Raitoharju et al. [36] reported that miR-484 is negatively correlated with extra-large VLDL phospholipid levels and early insulin resistance-related metabolites.

In our study, fruit intake frequency, including whole fruit and homemade 100% fruit juices, was significantly associated with the risk of T2DM, with daily fruit consumption being an independent protecting factor over occasional fruit intake. This result would be in line with previous literature supporting the relationship between fruit consumption and risk of developing T2DM [22,23,24], as well as the therapeutic role of fruit in preventing the onset of T2DM and its complications. Research suggests that clinical improvement in patients with T2DM can be achieved by increasing antioxidant defences [19]; in this sense, complementary therapies based on the use of natural antioxidant and anti-inflammatory compounds, such as Pycnogenol, a French pine bark extract containing procyanidins with strong antioxidant potential, have been proposed as novel approaches to the management of various chronic diseases [49]. Fruit is an important natural source of antioxidant and anti-inflammatory compounds, as well as other phytochemicals that play a critical role in diabetes management, glycemic control and the development of associated complications through various pathways and mechanisms, such as enhancing beta-cell function, improving insulin sensitivity, inhibiting carbohydrate-digesting enzymes, reducing glucose production in the liver, or reducing inflammation and oxidative stress, among others [10,15,19]. Preclinical and clinical evidence has demonstrated the benefits of inflammation-targeted dietary therapies using phytocomponents present in fruit in the treatment of hyperglycemia, β-cell dysfunction, and insulin resistance in diabetes [12,13,14]; in addition, cohort studies have shown that higher fruit intake is associated with a reduced risk of T2DM [17,18,22,23], with evidence of a non-linear relationship [24]. However, controversial results include associations between fruit juice and the risk of T2DM. A recent review of existing prospective evidence suggested that fruit juice consumption is not associated with T2DM [50], whereas other authors suggest that higher fruit juice consumption is associated with a higher risk of T2DM [17,51]. The weakness of dietary assessment questionnaires to distinguish between natural 100% fruit juice and other sources of fruit juice, which may include commercial fruit drinks with added sugar and very little fruit, has been proposed as the main reason for these discrepancies [50]. To avoid this limitation, in our study, homemade 100% fruit juice was considered separately from other sources of fruit juice or sugared fruit beverages; however, due to the inclusion of fruit juice consumption alongside whole fruit intake in the definition of fruit intake frequency, we are not able to distinguish the independent effect of each on T2DM risk.

Several authors have proposed that specific nutrients or dietary factors may modulate the miRNA expression in different cells or tissues related to obesity, T2DM and other metabolic diseases, with subsequent effects on metabolism [29,30,31]. There is increasing evidence that phytochemicals present in fruit, such as polyphenols, flavonoids, carotenoids or alkaloids, have the potential to regulate the expressions of different miRNAs in vivo and in vitro [52,53,54,55]; however, the effects of these phytochemical components on miR-484 levels have been less explored. In this study, we also aimed to investigate the possible relationship between miR-484 levels and fruit consumption and its implications for the development of T2DM. Our results showed that miR-484 levels were not associated with the frequency of fruit intake. No previous data on the nutritional regulation of miR-484 levels by fruit intake have been reported to date. To our knowledge, only one study has focused on the regulation of miR-484 levels, using an animal model and focusing on the role of the phenolic components of olive oil [56]. It showed that the brain tissue of mice fed with extra-virgin olive oil rich in phenolic compounds had higher miR-484 expression levels compared to control animals fed with the same olive oil deprived of phenolic components, suggesting that miR-484 expression levels could be modulated by phenolic components. Unlike the study by Luceri et al., this study analysed circulating serum miR-484 levels. As miRNA present in bio-fluids could originate from different tissue sources, their circulating levels do not necessarily correlate with their tissue miRNA expression; thus, various studies have reported differences between circulating and tissue miRNA levels [57,58].

In our study, besides the multiplicative interaction effect found between miR-484 categories and fruit frequency consumption on T2DM risk, the additive interaction indicators RERI and AP also point to a positive additive interaction. The presence of an additive interaction is especially relevant for prevention and public health in general and an additive scale is proposed to more closely correspond to a mechanistic interaction [45]; in this regard, our interaction analysis suggests not only a synergistic, but also a multiplicative interaction between low miR-484 levels and occasional fruit intake towards a significant increase in the likelihood of incident T2DM. In general, it is common that two factors with a multiplicative interaction also present a positive interaction in the additive model [59]; considering this, the results of our multiplicative and additive interaction models are essentially consistent with each other in the nature of biological interactions. Altogether, our results suggest that fruit intake frequency modifies of the effect of miR-484 levels on T2DM development on both multiplicative and additive scales, and demonstrate that the combination of low miR-484 levels and occasional fruit intake may significantly increase the likelihood of T2DM compared to their independent effects. These findings are noteworthy, as there are no previous studies investigating this interaction or the biological mechanisms underlying it. A relevant role of miR-484 in the regulation of mitochondrial dysfunction by targeting mitochondrial fission 1 protein has been established [37,38]. Reshma et al. reported a down-regulation of mitochondrial fission 1 protein in ischemic induced H9c2 cells by the pre-treatment with T. terrestris L. fruit methanol extract [60], suggesting that phytocomponents of T. terrestris L. fruit could protect mitochondrial function in H9c2 cells. Alternatively, several authors have proposed the beneficial antioxidant effects of some fruits and their biocomponents on T2DM development through the control of different pathways involved in mitochondrial dysfunction, such as mitochondrial fission [8,12,14,61]. Considering all this, it is reasonable to speculate the possibility that miR-484 and some natural biocomponents of fruit have an interplaying role in regulating mitochondrial function, which contributes to enhanced individual effects on their association with the risk of developing T2DM. Further studies are warranted to explore this relationship and the underlying mechanisms.

The main strength of this research is that the data were obtained from a sizable nationwide cohort, with a considerable follow-up duration and a substantial number of events. Most participants underwent an OGTT, and HbA1c was also considered in the follow-up, which assures the capture of most incident T2DM. The physical activity of the participants was assessed using a widely standardised test, and detailed dietary information was collected. The study reported results of multiplicative and additive interactions, which strongly supported the conclusions. Our study also presents some limitations. Although rate of follow-up participation was 66% and the possible participation bias in the whole study was minimal [40], the influence of some confounding factors cannot be ruled out. Differences due to interventions carried out in the participants’ area of origin were not specifically assessed; however, all the procedures were performed by the same trained nurses both at baseline and during follow-up, so data and sample collection are expected to have less variability. It is not possible to know what the portion size or the total amount of fruit consumed were in this study. However, evidence suggests that there is a non-linear relationship between the amount of fruit consumed and the risk of T2DM, namely, that the risk reduction associated with optimal fruit consumption is up to 200–300 g/day compared to non-consumption. No benefit is apparent for increasing intake above this value [24]. An important limitation of this study is the lack of information on the type of fruit consumed. Sugar content varies among different types of fruit, and this can affect glucose metabolism differently and lead to a different glycemic response. Nevertheless, despite the potentially injurious effect of fruit sugar content on T2DM risk, most of the current preclinical and clinical evidence is consistent with the preventive role of other fruit natural components such as dietary fiber, antioxidants, vitamins, minerals and several phytochemical components [11,12,13,14], which are present in all the commonly consumed fruit regardless of the type of fruit. On the other hand, most of the fruit consumed in Spain has a low glycemic index, such as citrus fruits, apples or pears; while other fruits with a higher glycemic index, such as mangoes or custard apples, are consumed more sparingly. However, although the glycemic index of some fresh fruit could be high, the glycemic load of virtually all fresh fruit is very low; thus, the glycemic load is always low if the fruit is fresh. In addition, as previously mentioned, the consideration of whole fruit and natural 100% fruit juice consumption together in the definition of the variable fruit frequency intake hinders us in differentiating each independent effect on T2DM risk. Finally, the tissue origin or destination of circulating miR-484 are unknown in this study; however, differences in its levels associated with incident T2DM are enough to propose it as biomarker for the future risk of T2DM.

## 5. Conclusions

In conclusion, our study in a Spanish representative cohort demonstrated an independent protecting effect of daily fruit intake and elevated miR-484 levels on the risk of incident T2DM. Our findings have also pointed to a positive interaction between miR-484 levels and fruit intake frequency in relation to incident T2DM, which indicated that the combined effect of low miR-484 levels and occasional fruit intake could significantly increase the risk of T2DM compared to their independent effects alone. Altogether, our results support the nutritional recommendations of daily fruit consumption and reinforce the idea that fruit can be used as a preventive therapy for the metabolic disturbances involved in T2DM, perhaps through an interconnected effect with miR-484 on the control of mitochondrial function. Further investigations with rigorously designed clinical trials are prerequisite to confirm the associations and to explore the underlying mechanisms.

## Figures and Tables

**Table 1:** General clinical characteristics according to the miR-484 categories.

	Overall Population	Low miR-484	High miR-484	*p*-Value
	(n = 2234)	(n = 555)	(n = 1679)	
Age (%)				0.1
18–30 years	12.5	12	13	
31–45 years	33.2	31	34	
46–60 years	31.8	30	32	
61–75 years	19	23	18	
>75 years	3.5	4	3	
Sex (%men)	39.2	38.2	39.5	0.61
BMI (Kg/m^2^)	27.56 ± 4.73	26.98 ± 4.45	27.76 ± 4.81	<0.001
Fasting serum glucose (mg/dL)	91.69 ± 11.84	89.79 ± 12.08	92.31 ± 11.70	<0.001
Fasting serum insulin (mU/dL)	8.52 ± 5.31	7.70 ± 3.94	8.80 ± 5.67	0.01
Systolic blood pressure (mmHg)	128.67 ± 18.47	129.04 ±19.22	128.55 ± 18.22	0.58
Diastolic blood pressure (mmHg)	76.34 ± 10.30	76.34 ± 10.21	76.42 ± 10.42	0.68
Total cholesterol (mg/dL)	197.78 ± 38.84	196.75 ± 37.82	198.12 ± 39.18	0.24
HDL cholesterol (mg/dL)	53.01 ± 12.99	53.13 ± 12.31	50.97 ± 13.21	0.25
LDL cholesterol (mg/dL)	106.26 ± 29.30	105.61 ± 28.31	106.47 ± 29.62	0.5
HOMA index	1.98 ± 1.41	1.75 ± 1.01	2.06 ± 1.51	<0.01
Prediabetes (%)	27.8	23.2	29.3	<0.01
Smoking (%current smoker)	26.5	26.1	25.4	0.79
Alcohol consumption (%)				0.06
Never	23.9	26.8	22.9	
Low	10.1	8.1	10.7	
Moderate	50	51	49.7	
High	16	14.1	16.6	
Educational level				<0.001
Unlettered	8	5.9	8.6	
Primary/High School	74.2	70.6	75.4	
University	17.8	23.4	16	
SF-IPAQ score				0.68
Low (%)	42.4	41.3	42.8	
Moderate (%)	34.2	35.7	33.7	
High (%)	23.4	23.1	23.6	
Fruit intake (%Daily)	75.4	77.5	74.7	0.21

Data presented as Mean ± standard deviation, or proportions. Significant differences according to the miR-484 categories were measured by ANOVA adjusted for sex, age and BMI or Chi-Square test. BMI: body mass index; HDL: high-density lipoprotein; LDL: low-density lipoprotein; HOMA: homeostasis model assessment; SF-IPAQ score: Short Form of International Physical Activity Questionnaire Score.

**Table 2:** Multivariable association analysis for fruit intake frequency and miR-484 categories with the risk of incident T2DM.

	M1		M2	
	OR (95%CI)	*p*	OR(95%CI)	*p*
Age (years)				
18–30	RC		RC	
31–45	5.61 (1.57–36.21)	0.02	5.36 (1.49–34.68)	0.03
46–60	8.18 (2.3–52.9)	*p* < 0.01	7.9 (2.2–51.27)	*p* < 0.01
61–75	15.1 (4.18–98.25)	*p* < 0.001	14.58 (3.96–95.57)	*p* < 0.001
>75	21.89 (4.98–156.2)	*p* < 0.001	19.69 (4.27–144.04)	*p* < 0.001
Sex (woman vs. male)	0.74 (80.5–1.08)	0.12	0.75 (0.49–1.16)	0.20
BMI	1.08 (1.03–1.12)	*p* < 0.001	1.07 (1.03–1.12)	*p* < 0.001
Fasting glucose level	1.07 (1.05–1.09)	*p* < 0.001	1.07 (1.04–1.09)	*p* < 0.001
Family history of T2DM (yes vs. no)	2.13 (1.43–3.2)	*p* < 0.001	2.11 (1.41–3.19	*p* < 0.001
HOMA levels	1.14 (1.01–1.28)	0.03	1.15 (1.01–1.29)	0.03
Fruit intake (Daily vs. occasionally)	0.28 (0.13–0.64)	*p* < 0.01	0.28 (0.13–0.65)	*p* < 0.01
miR-484 levels (High vs. Low)	0.29 (0.13–0.67)	*p* < 0.01	0.28 (0.12–0.63)	*p* < 0.01
Fruit intake*miR-484 levels	2.86 (1.10–7.35)	0.03	2.89 (1.11–7.48)	0.03
Smoking habits (current vs. never/former)			1.02 (0.62–1.66)	0.94
Educacional level				
Unlettered			RC	
Primary/High School			0.79 (0.45–1.44)	0.43
University			0.55 (0.24–1.25)	0.16
Alcohol consumption (%)				
Never			RC	
Low			1.09 (0.49–2.28)	0.82
Moderate			1.02 (0.63–1.7)	0.93
High			1.01 (0.54–1.87)	0.99
SF-IPAQ score				
Low (%)			RC	
Moderate (%)			1.36 (0.88–2.09)	0.16
High (%)			0.95 (0.54–1.63)	0.87

RC: Reference category; OR: Odd ratio; CI: Confidence interval. M1: Logistic regression model for the risk of T2DM incidence adjusted by sex, age, BMI, fasting serum glucose, family history of T2DM, insulin resistance index and interaction term miR-484xFruit intake. M2: M1+ lifestyle variables (smoking habits, educational level, alcohol consumption and physical activity). BMI: body mass index; HOMA: homeostasis model assessment. SF-IPAQ score: Short Form of International Physical Activity Questionnaire Score.

**Table 3:** Additive interaction analysis between miR-484 categories and frequency of fruit consumption on T2DM development at follow-up.

		miR-484 Categories		
		High	Low	ORs (95%CI) for miR-484_Low Within Strata of Fruit Consumption
		OR (95%CI)	OR (95%CI)	
**-** **M1**				
Fruit consumption	Daily	RC (1)	1.2 (0.72, 2)	1.2 (0.72, 2)
	Occasional	1.25 (0.74, 2.12)	4.3 (2.09, 8.86)	3.43 (1.53, 7.7)
ORs (95%CI) for occasional fruit consumption within strata of miR-484 categories		1.25 (0.74, 2.12)	3.58 (1.6, 8.03)	
RERI (95%CI)			2.85 (0.56, 7.28)	
AP (95%CI)			0.66 (0.11, 0.82)	
SI (95%CI)			7.3 (0.83, 64.36)	
**-** **M2**				
Fruit consumption	Daily	RC (1)	1.25 (0.74, 2.09)	1.25 (0.74, 2.09)
	Occasional	1.22 (0.72, 2.09)	4.41 (2.13, 9.12)	3.61 (1.6, 8.13)
ORs (95%CI) for occasional fruit consumption within strata of miR-484 categories		1.22 (0.72, 2.09)	3.54 (1.57, 7.96)	
RERI (95%CI)			2.94 (0.6, 7.52)	
AP (95%CI)			0.67 (0.12, 0.82)	
SI (95%CI)			7.27 (0.87, 60.85)	

OR: Odd ratio; CI: Confidence interval; RERI: relative excess risk due to interaction; AP: attributable proportion due to interaction. M1: Logistic regression model for the risk of T2DM incidence adjusted by sex, age, BMI, fasting serum glucose, family history of T2DM, insulin resistance index and interaction term miR-484xFruit intake. M2: M1+ lifestyle variables (smoking habits, educational level, alcohol consumption and physical activity).

## Data Availability

The datasets presented in this article are not readily available because they are part of an ongoing study. Requests to access the datasets should be directed to the corresponding authors upon reasonable request.
